# Evidence of Immunoproteasome Expression Onset in the Formative State of Pluripotency in Mouse Cells

**DOI:** 10.3390/cells13161362

**Published:** 2024-08-15

**Authors:** Daria Kriger, Uliana I. Podenkova, Evgeny I. Bakhmet, Evgenii Potapenko, Elena Ivanova, Alexey N. Tomilin, Anna S. Tsimokha

**Affiliations:** 1 Institute of Cytology, Russian Academy of Sciences, Tikhoretsky Ave. 4, 194064 St. Petersburg, Russia; dkriger@incras.ru (D.K.); uliana.podenkova@gmail.com (U.I.P.); e.bakhmet@incras.ru (E.I.B.); a.tomilin@incras.ru (A.N.T.); 2 Institute of Evolution, University of Haifa, Haifa 3498838, Israel; potapgene@gmail.com

**Keywords:** differentiation, embryonic stem cells (ESCs), epiblast-like cells (EpiLCs), ubiquitin–proteasome system, immunoproteasome, Lmp7/Psmb8, Lmp2/Psmb9, Mecl1/Psmb10, naïve, formative and primed states of pluripotency

## Abstract

Embryonic stem cells (ESCs) are remarkable for the high activity level of ubiquitin–proteasome system—the molecular machinery of protein degradation in the cell. Various forms of the proteasome complexes comprising different subunits and interacting regulators are responsible for the substrate selectivity and degradation. Immunoproteasomes are amongst these forms which play an important role in antigen presentation; however, a body of recent evidence suggests their functions in pluripotent stem cells. Previous studies have established three consecutive phases of pluripotency, featured by epiblast cells and their cultured counterparts: naïve, formative, and primed phase. In this work, we report that immunoproteasomes and their chaperone co-regulators are suppressed in the naïve state but are readily upregulated in the formative phase of the pluripotency continuum, featured by epiblast-like cells (EpiLCs). Our data lay ground for the further investigation of the biological functions of immunoproteasome in the regulation of proteostasis during early mammalian development.

## 1. Introduction

Embryonic stem cells (ESCs), isolated from pre-implantation blastocysts, are pluripotent cells that possess an unlimited self-renewal capacity and the ability to differentiate into all types of somatic cells [1]. It is known that ESCs have an increased resistance to genome damage and reduced frequency of mutations, and produce much less oxygen radicals than differentiated cells [2,3]. Moreover, ESCs, presumably, have the ability to timely eliminate proteotoxic factors that could lead cells to exit from the pluripotent state and/or promote aging.

The ubiquitin proteasome system (UPS) carries out most of the regulated proteolysis in the cell and plays a crucial role in many cellular processes. Currently, the term “proteasome” covers the entire family of protein complexes that have a common proteolytic core, the 20S core particle, but differ in the proteasome activators associated therewith [4,5]. Under certain conditions, such as exposure to various cytokine, the constitutive catalytic subunits of the proteasome can be replaced by inducible ones. In this configuration, the proteasome, referred to as immunoproteasome, participates in the immune response, processing antigens for their presentation by MHC-I [6,7]. The stimulation of cells with γ-interferon (IFNγ) induces not only the expression of immunoproteasome subunits genes, but also upregulates the levels of alpha and beta subunits of the PA28 regulatory complex, which, via binding to the core particle of the proteasome, increases its ability to degrade small peptides [8].

Pluripotency—an intrinsic ability to generate all cell lineages of an organism—exists in three forms: naïve, formative, and primed. These forms correspond to natural consecutive states of epiblast cells at the late blastocyst, early post-implantation, and gastrulation stages, respectively. Moreover, the three pluripotency states are recapitulated by cultured counterparts of epiblast cells: ESCs, epiblast-like cells (EpiLCs), and epiblast stem cells (EpiSCs), respectively (reviewed in [9,10]). These cell types, often interconvertible in culture, serve as a convenient in vitro model for dissecting molecular events controlling differentiation from naïve epiblast to the three germ layers (ectoderm, endoderm, and mesoderm) via the formative and primed epiblast states.

Over recent years, a line of evidence pointed to an important role of immunoproteasomes in the induction and maintenance of pluripotency, as well as in the exit from this cellular state through differentiation [11,12,13,14,15,16]. It was found that immunoproteasome subunits are abundant in human ESCs, while their expression decreases as the cells proceed to differentiation [17]. Using inhibitors of immunoproteasome subunits, it was shown that the suppression of immunoproteasome activity led to a downregulation of pluripotent markers and an upregulation of differentiation markers [17]. At the same time, mouse ESCs showed detectable immunoproteasome subunit expression only by the second day of differentiation [18]. This discrepancy can be explained by different pluripotent states of cultured mouse and human ESCs [10,19]. Mouse ESCs proceed all the way from naïve pluripotency to terminal differentiation via the formative state, which is reached by the second day of the differentiation in culture, whereas human ESCs are already in the primed state corresponding to that of mouse EpiSCs [20]. It should be noted that there are a number of studies in which progress has been made in converting cultured human ESCs to a naïve state [21,22].

In this study, we have undertaken a detailed analysis of immunoproteasome and accessory chaperone expression during the transition from the naïve to the formative state of pluripotency, featured by mouse ESCs and EpiLCs, respectively.

## 2. Materials and Methods

### 2.1. Cell Culture

Murine E14 Tg2a ESCs (BayGenomics, Berkeley, CA, USA) in a metastable naïve state of pluripotency (referred to hereafter as ESCs) were cultured in the SL (serum-LIF) medium comprising Knockout DMEM (ThermoFisher, Waltham, MA, USA), 15% fetal bovine serum (FBS, HyClone, GE Healthcare Life Sciences, Marlborough, MA, USA), penicillin 100 U/mL/streptomycin 100 μg/mL (ThermoFisher), 2 mM l-glutamine (ThermoFisher), 1X non-essential amino acids (NEAA, ThermoFisher), and 100 μM β–mercaptoethanol (Sigma-Aldrich, Darmstadt, Germany), and supplemented with 500 units/mL of leukemia inhibiting factor (LIF, produced in the laboratory) under standard conditions (37 °C, 5% CO_2_) on feeder-free gelatin-coated culture plates.

To obtain ESCs in the ground naïve state of pluripotency (referred to hereafter as naïve ESCs), the ESCs were cultured in the 2iL medium: N2B27 medium supplemented with 3 μM CHIR99021 (Sigma-Aldrich), 1 μM PD0325901 (Sigma-Aldrich), and LIF for 5 days on plastic treated with 0.01% poly-L-ornithine solution (Sigma-Aldrich). Cells were passaged using 0.05% Trypsin/EDTA solution (ThermoFisher). The N2B27 medium comprised DMEM/F-12 (ThermoFisher) and Neurobasal medium (ThermoFisher) in a 1:1 ratio, N2 (ThermoFisher) and B27 supplements (ThermoFisher), 50 μM β-mercaptoethanol (Sigma-Aldrich), 2 mM L-glutamine (ThermoFisher), 100 mg/mL penicillin-streptomycin, and 0.005% bovine serum albumin (Sigma-Aldrich).

Cells were passaged using 0.05% Trypsin/EDTA solution (ThermoFisher).

The EpiLCs were derived by transferring the naïve ESCs onto fibronectin-coated plates (10 μg/mL, Merck, Darmstadt, Germany) and culturing in the EpiLC medium (N2B27 medium supplemented with 20 ng/mL Activin A (Peprotech, London, UK)), 12 ng/mL fibroblast growth factor 2 (bFGF, Peprotech), and 1% serum replacement (KnockOut Serum Replacement, ThermoFisher) for 48 h.

### 2.2. Differentiation of ESCs

ESC differentiation was induced in the SL medium by LIF withdrawal and, when indicated, 0.1 µM of retinoic acid (RA, Sigma-Aldrich) was added, following the previously established protocol [23].

### 2.3. Immunocytochemistry

The cells were fixed on culture plates with a solution of 4% paraformaldehyde (Sigma-Aldrich) at room temperature for 15 min, then washed with PBS and permeabilized using a 0.1% Triton X-100 solution (AppliChem, Darmstadt, Germany) for 15 min at room temperature. Fixed cells were incubated for an hour at room temperature in 3% BSA blocking solution (neoFroxx, Einhausen, Germany) diluted in PBST. Then, overnight at a temperature of +4 °C, incubation was carried out with primary antibodies (Table S1) diluted in PBST buffer containing 0.3% BSA. The next day, the cells were washed 5 times with PBST, incubated with secondary fluorescent antibodies for 3 h at room temperature, washed 3 more times with PBST solution, and counterstained with DAPI (Sigma-Aldrich) diluted in PBST (1:5000) for 5 min at room temperature, after which the solution was replaced with PBST with the addition of sodium azide. The results of the staining were documented using an EVOS FL AUTO fluorescence microscope (ThermoFisher).

### 2.4. RNA Extraction and qPCR Analysis

Total RNA was extracted using Extract RNA reagent (Evrogen, Moscow, Russia) according to manufacturer’s instructions and treated with DNaseI (ThermoFisher). The quality and concentration of extracted RNA samples were measured using a NanoDrop 2000 spectrophotometer (ThermoFisher). cDNA was synthesized using a RevertAid RT kit (ThermoFisher) or a MMLV RT kit (Evrogen) with random or oligo(dT) primers.

The quantitative polymerase chain reactions (qPCR) were performed with qPCRmix-HS SYBR (5×) mix (Evrogen) or Biomaster HS-qPCR SYBR Blue (2×) mix (Biolabmix, Novosibirsk, Russia) and gene-specific primers (Table S2) on an Applied Biosystems 7300 Real-Time PCR System. The relative quantification of target genes was calculated using the 2^−ΔΔCt^ method with the Gadph and beta-2-microglobulin used as the reference genes [24,25]. The absence of non-specific amplification and dimer formation of primers was checked by setting negative controls without a cDNA template and analyzing the melting curves of the amplification products.

### 2.5. Western Blot Analysis

Cells were lysed in buffer A (100 mM NaCl, 50 mM Na_2_PO_4_, pH 7.5, 10% glycerol, 5 mM ATP, 1 mM DTT, 5 mM MgCl_2_, ×1 protease inhibitors, 0.5% NP-40) [26] for 30 min at +4 °C, then cell lysates were sonicated on a Fisher Scientific Sonicator CL-18 for 5 cycles of 10 s with an amplitude of 50%. The protein concentration in samples was determined by the Bradford method at a wavelength of 595 nm. The samples were then separated under denaturing (SDS) conditions on 13%-polyacrylamide gel electrophoresis. Transfer to a PVDF membrane (Bio-Rad Laboratories, Hercules, CA, USA) was performed using semi-dry blotting at 20 V, 1 mA/cm^2^ for 120 min. The membrane was blocked in a solution of 3% BSA (BioFroxx) in PBST for 50 min. Then, the membrane was incubated with primary antibodies (Table S1) overnight at 4 °C. The next day, the membrane was washed with PBST 3 times for 10 min, and incubated with secondary antibodies at room temperature for 1 h. We used anti-rabbit (GAR-HRP), anti-mouse (GAM-HRP), or anti-guinea pig (GAGP-HRP) (1:5000, Jackson Immunoresearch, West Grove, PA, USA) secondary antibodies. To detect proteins bound to antibodies, SuperSignal Femto (ThermoFisher) was applied to the membrane and the chemiluminescent signal was recorded using the ChemiDoc Touch Imaging System (Bio-Rad Laboratories).

### 2.6. Statistical Analysis

Statistical analysis was performed using the SigmaPlot 12.0 (Systat Software Inc., San Jose, CA, USA). Differences between groups were analyzed using one-way ANOVA with Tukey’s post-hoc test. *p*-Values < 0.05 were considered as significant.

## 3. Results

In contrast to human ESCs, which are in the primed state of pluripotency, mouse ESCs are in the naïve state of pluripotency [20]. However, when cultured in the presence of serum and LIF (referred to as SL medium), the latter cells shown heterogeneity, demonstrating some features of primed pluripotency, and thus are designated as metastable naïve ESCs [27].

**Figure 1:**
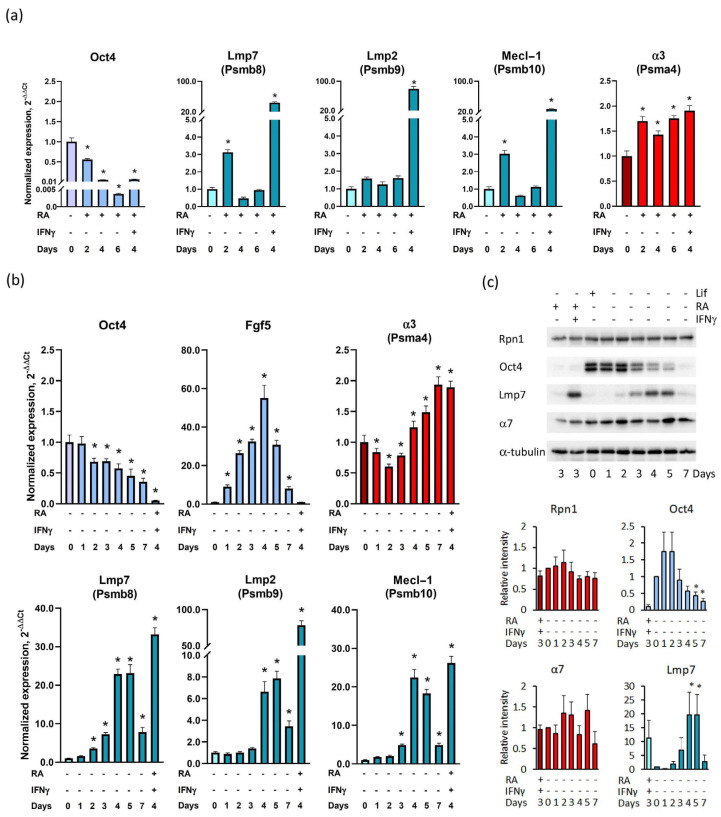
Mouse ESC differentiation induced by RA addition and/or LIF withdrawal is accompanied by the upregulation of immunoproteasome subunits. (**a**) Relative levels of Oct4, Lmp7 (Psmb8), Lmp2 (Psmb9), Mecl-1 (Psmb10), α3 (Psma4), and Gapdh mRNAs (the latter is used as a reference mRNA) in ESCs during differentiation induced by retinoic acid (RA) addition and LIF withdrawal. (**b**) Relative levels of the above as well as of the primed pluripotency marker mRNA Fgf5 in ESCs during differentiation promoted solely by LIF withdrawal. (**c**) Representative image of Western blot analysis of Rpn1 (19S subunit), 20S core subunit α7, immunoproteasome subunit Lmp7, and into pluripotency marker Oct4 in undifferentiated ESCs (set as day 0) and cells undergoing differentiation induced by LIF withdrawal (above panel). Quantification of the above results by measuring Western blot band intensities using the Image Lab 6.0.1 Software (Bio-Rad Laboratories) is shown in the charts below. Cell lysates of ESCs treated for 3–4 days with RA or treated for 2 days with RA and, additionally, for 1–2 days with IFNγ (as indicated below each chart) were used as controls. Values are shown as mean ± s.e.m. of at least three independent experiments. Significant values are indicated as * *p* < 0.05 (one-way ANOVA followed by Tukey’s post hoc test).

We first assessed the dynamics of immunoproteasome gene expression during mouse ESC differentiation. ESCs were routinely maintained under the SL condition and then induced to differentiate by the addition of RA and LIF withdrawal. Marker mRNA levels were evaluated by qPCR on days 0, 2, 4, and 6 of the differentiation. As expected, the levels of the pluripotency factor Oct4 mRNA had already decreased by day 2 (Figure 1a). On the contrary, we observed a statistically significant increase in the relative levels of mRNA of the immunoproteasome subunits Lmp7 (Psmb8) and Mecl-1 (Psmb10) mRNAs on day 2. However, upon comparison, Lmp7, mp2 (Psmb9), and Mecl-1 levels reached in ESCs upon on day 2 of differentiation were noticeably lower than those in ESCs treated for 2 days with RA and, additionally, for 2 days with RA and IFNγ (Figure 1a).

We next examined the presence of immunoproteasome subunit mRNAs during ESC differentiation induced solely by LIF withdrawal, following the previously established protocol [23]. As demonstrated by qPCR, Oct4 mRNA expression gradually decreased and immunoproteasome-specific subunit Lmp7, Lmp2, and Mecl-1 mRNA levels dramatically increased by days 4–5 and then decreased by day 7 of the differentiation. As a control of immunoproteasome induction with IFNγ, we used ESC-derived differentiated cells generated by LIF withdrawal and treatment with RA for 2 days, followed by 2-day treatment with IFNγ. Notably, the transcript of the 20S proteasome subunit α3 (Psma4) gradually increased (about 2-fold) by day 7 of the differentiation (Figure 1b). However, the Western blot analysis showed that the expression of the 20S proteasome subunit α7 and 19S regulatory subunit Rpn1 was stably maintained throughout day 7 of the differentiation (Figure 1c). In contrast, the protein level of the immunoproteasome subunit Lmp7 markedly increased by days 4–5 and then decreased by day 7 of the differentiation. As a control of Lmp7 induction with IFNγ, we used cell lysates of ESC-derived differentiated cells generated by LIF withdrawal and treatment with RA for 2 days, followed by 1-day treatment with IFNγ. The pan-pluripotent (i.e., non-specific for either naïve, formative, or primed states) marker Oct4 mRNA content significantly declined by day 7. It should be also noted that the primed pluripotency marker Fgf5 expression increased gradually by day 4, then declined by day 7 of the differentiation (Figure 1b). Taken together, these data suggest that ESCs, deprived of LIF, proceed to terminal differentiation via the formative and/or primed pluripotency states, accompanied by the onset of immunoproteasome expression. This conclusion is further substantiated by the analysis of publicly available single-cell RNA-seq datasets [28,29], which demonstrates Psmb8 expression in the cell population annotated, according to marker expression, to be in the primed pluripotency state (Figure S1).

**Figure 2:**
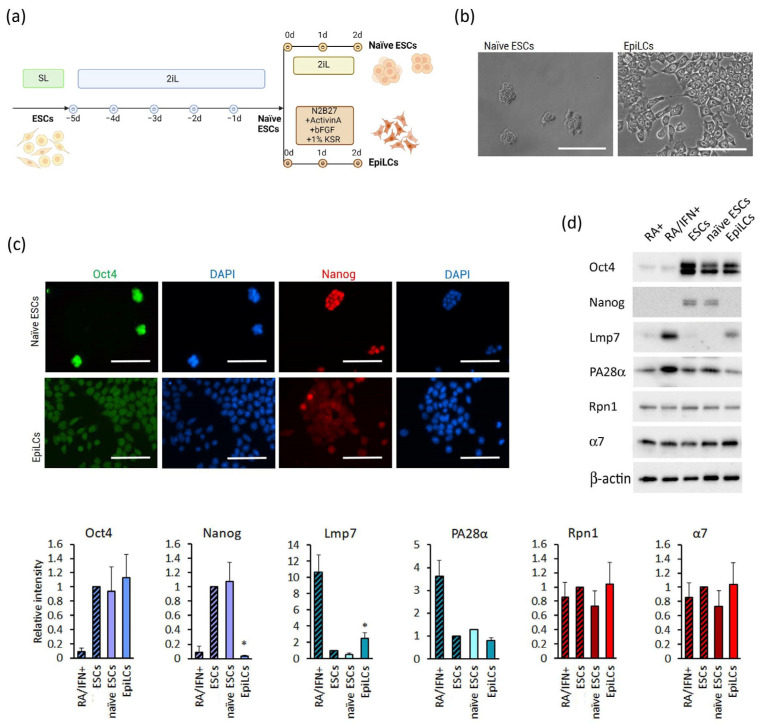
Transition of mouse ESCs from the naïve to the formative pluripotency state (the latter is featured by EpiLCs) induces the expression of immunoproteasome subunit Lmp7. (**a**) Schematic representation of EpiLC derivation protocol from ESCs: SL—standard serum-containing medium supplements with LIF, 2iL—serum-free medium containing two inhibitors (CHIR99021, PD0325901) and LIF. (**b**) Morphology of naïve ESCs (**left**) and derived from them EpiLCs (**right**), scale bar—100 µm. (**c**) Representative immunostaining of naïve ESCs (**upper panel**) and EpiLCs (**bottom panel**) using antibodies against Oct4 (in green) and Nanog (in red). Nuclei were counterstained with DAPI (blue). Scale bar—100 μm. (**d**) Representative Western blot analysis of immunoproteasome subunit Lmp7, PA28 regulator subunit α, 20S core subunit α7, 19S regulator subunit Rpn1, and the pluripotency markers Oct4 and Nanog in naïve ESCs and EpiLC lysates. ESCs treated for 3 days with RA or treated for 2 days with RA and, additionally, for 1 day with IFNγ served as a control. Band intensities were analyzed using the Image Software (Bio-Rad Laboratories) and presented in the charts below. Values are shown as mean ± s.e.m. of at least three independent experiments. Significant values are indicated as * *p* < 0.05 (one-way ANOVA followed by Tukey’s post hoc test).

To substantiate the above hypothesis, we purposely induced the differentiation of ESCs into EpiLCs, an intermediate cell type in the formative state of pluripotency, following the previously established protocol depicted in Figure 2a [30]. To this end, ESCs routinely maintained in standard SL medium were put into the ground state of naïve pluripotency by culturing in a serum-free N2B27 medium supplemented with GSK/MAPK inhibitors (2i) and LIF (referred to hereafter as 2iL medium) for 5 days. The maintenance under the 2iL medium is known to remove the heterogeneity of ESC cultures, promoting the homogeneous expression of naïve markers [27]. To induce the naïve-to-primed transition of pluripotency states, cells were then transferred to a medium containing bFGF and Activin A. Arising EpiLCs exhibited a typical flattened morphology after two days of culturing under the indicated conditions (Figure 2b). The pluripotency status of ESCs and EpiLCs was confirmed by immunocytochemical staining for Oct4 and Nanog (Figure 2c). Oct4 expression was retained, while Nanog declined in these cells, which is consistent with previously reported expression patterns of these two factors during the transition of pluripotency states [30].

We next assessed the expression of immunoproteasome subunits during the naïve-to-primed transition using the Western blot analysis (Figure 2d). As a positive control, we used ESCs treated for 2 days with RA in which the expression of immunoproteasome subunits was induced by 24 h IFNy treatment. As before, pluripotency status was affirmed by Oct4 and Nanog staining. We observed a dramatic decrease in the naïve marker Nanog expression along with the retention of Oct4 expression in EpiLCs, confirming the formative state of pluripotency in the latter cell type. Importantly, EpiLCs showed immunoproteasome subunit Lmp7 expression, whereas this subunit was absent from naïve ESCs (Figure 2d). Interestingly, PA28 regulator subunits α and β (Psme1 and Psme2, respectively) expressed uniformly across naïve ESCs and EpiLCs (Figure 2d and Figure S3a). It was also worth noting that 20S proteasome subunit α7 also remained unchanged, as expected. Finally, we did not observe changes in the expression level of 19S proteasome regulatory particle base subunit Rpn1. An analysis of a publicly available transcriptome dataset [30] demonstrated that the differentiation of the naïve ESCs into fully primed EpiSCs is accompanied by the expression of immunoproteasome subunit Lmp7 (Figure S2). However, our results indicate that immunoproteasome activation during the transition of mouse ESCs into the primed state already occurs at the formative (EpiLCs) stage.

**Figure 3:**
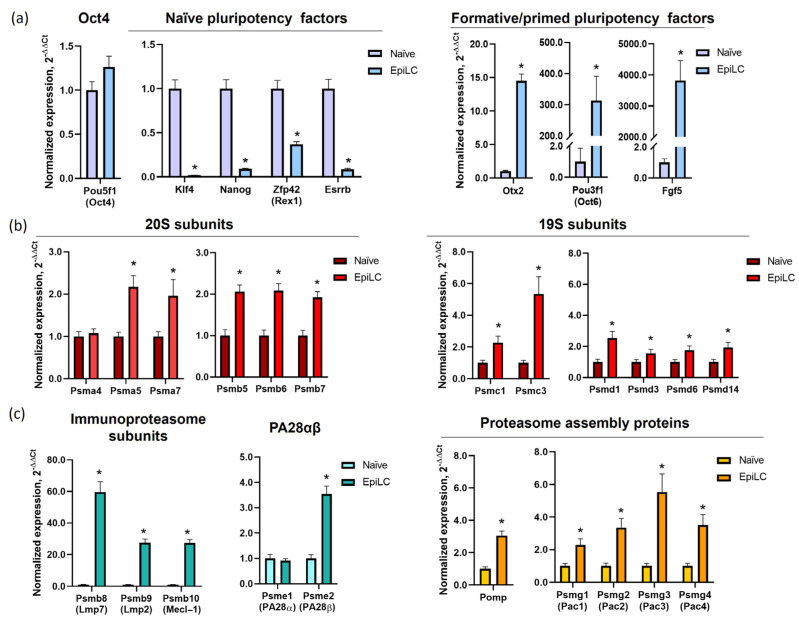
Upregulation of immunoproteasome subunits and accessory proteins in the EpiLCs, shown by qPCR. (**a**) Downregulation of the naïve pluripotency markers mRNAs Klf4, Nanog, and Zfp42 (Rex1), upregulation of primary pluripotency markers Otx2, Pou3fa (Oct6), and Fgf5, and unchanged levels of the pan-pluripotency marker Oct4 during the differentiation from naïve ESCs to EpiLCs. (**b**) Slightly increased expression levels of the constitutive 20S proteasome (**left**) and 19S regulatory subunits (**right**) upon the differentiation. (**c**) Significant upregulation of immunoproteasome subunits (Lmp7, Lmp2, and Mecl-1) and PA28 regulator subunits, as well as of the proteasome maturation protein Pomp and proteasome assembly chaperones Psmg1–4 in EpiLCs. Values are shown as mean ± s.e.m. of three independent experiments. Significant values are indicated as * *p* < 0.05 (one-way ANOVA followed by Tukey’s post hoc test).

To draw a more complete picture of immunoproteasome expression during the transition from naïve ESCs to EpiLCs, we performed a qPCR analysis. First, we assessed the expression of pluripotency markers to reaffirm the pluripotency state of ESCs and EpiLCs in our experimental setup. As expected, expression levels of the Oct4 mRNA in naïve ESCs and EpiLCs did not vary significantly. At the same time, the naïve pluripotency markers Klf4, Nanog, Zfp42 (Rex1), and Esrrb were dramatically downregulated in EpiLCs. On the contrary, Fgf5, Otx2, and Pou3f1 (Oct6), which are markers of the formative and primed states, have shown a dramatically upregulated expression in EpiLCs (Figure 3a). Thus, based on the expression patterns of the pluripotency markers, one can firmly assure the pluripotency states of the studied cells. We next proceeded to examine proteasomal subunit expression in these cells. As expected, the expression levels of 20S proteasome and 19S regulator subunits were slightly increased in EpiLCs compared to naïve ESCs (Figure 3B). We attribute this observation to an increased proliferative rate of EpiLCs compared to naïve ECSs (Figure S3b). In contrast, we revealed the dramatically increased expression of the immunoproteasome subunits Lmp7, Lmp2, and Mecl-1 (Figure 3c), whereas the expression of the PA28 regulator subunit mRNAs showed no (PA28α) or rather moderate (PA28β) upregulation in EpiLCs, albeit the latter protein showed unchanged expression in these cells (Figure S3a).

The formation of immunoproteasome is known to be assisted by proteasome assembly chaperones, or Pac’s [31]. Yet another chaperone serving this purpose is proteasome maturation protein (Pomp), which functions as a disposable chaperone [32]. Importantly, immunoproteasomes are assembled about four times faster and exhibit significantly reduced stability compared to constitutive proteasomes. Therefore, the onset of immunoproteasome function should presumably be accompanied by an upregulation of these chaperones. Indeed, we detected increased levels of Pac1 (Psmg1), Pac2 (Psmg2), Pac3 (Psmg3), Pac4 (Psmg4), and Pomp mRNA during the differentiation of naïve ESCs to the EpiLC (Figure 3c). Overall, our results support the hypothesis that the transition from the naïve to primed state of pluripotency is accompanied by the establishment of immunoproteasomes functions already at the formative pluripotency stage.

## 4. Discussion

The immunoproteasomes play a critical role in the immune system because they degrade intracellular proteins, including those of viral origin, into short peptides for their presentation by MHC-I molecules. It is important to note that constitutive proteasomes also perform this task [33]; however, the immunoproteasome has been shown to cleave proteins more efficiently than its constitutive counterparts [34] and to generate peptides that are better suited for binding to MHC-I molecules [35]. During recent years, a growing body of evidence suggests that immunoproteasomes are not solely involved in peptide presentation to the immune system, but also fulfill functions unrelated to antigen processing, including cytokine secretion and T-cell differentiation, as summarized elsewhere [36]. Infected or inflamed tissues as well as lymphoid organs have been found to express immunoproteasomes which mediate their function at various levels, including T-cell selection and epitope production, and thus form the protective CD8+ T cell responses to intracellular pathogens. Moreover, it can be assumed that the dysfunction of the immunoproteasome may contribute to autoimmune diseases in which CD8+ T cells play a central role [36]. Also, there is a disease spectrum currently known as proteasome-associated autoinflammatory syndromes (PRAAS), which is associated with specific mutations in the β5i/LMP7 (PSMB8) gene or other proteasome genes restricting proteasome function [36]. In this report, we provide compelling arguments that immunoproteasomes may play yet another biological function which is involved in epiblast development through the formative pluripotency state.

In agreement with previous studies [18], we observed a transient increase in immunoproteasome subunit expression during the first days of differentiation. Moreover, the upregulation of immunoproteasome subunit Lmp7 did not seem to be significant during the RA-induced differentiation of ESCs. In the SL medium deprived of LIF, the level of Lmp7 protein was elevated by days 4–5 of differentiation. Importantly, the immunoproteasome expression curve coincided with an increase in the expression of formative/primed pluripotency marker Fgf5 (Figure 1), which also supports the activation of immunoproteasomes in the state of formative pluripotency. Interestingly, a transcriptome dataset of human ESCs cultured at naïve and primed state of pluripotency also showed the expression of immunoproteasome subunit LMP7 in primed EpiSCs, but not in naïve ESCs [21], pointing to a common mechanism of immunoproteasome activation and similar roles of immunoproteasomes during embryogenesis in both species.

It remains to be determined what purpose immunoproteasomes serve during the transient formative pluripotency phase, featured by early post-implantation epiblast and its cultured counterpart, EpiLCs. Clearly, functional genetics must help to address this question. However, one may already speculate that immunoproteasome function involves a rapid turnover of specific factors during the transition of naïve cells into the primed state. The single and dual knockdown of genes, encoding immunoproteasome subunits LMP7 and LMP2 in human ESCs, resulted in either very minor changes in the expression of pluripotency and differentiation markers or no changes at all [17]. At the same time, the treatment of human ESCs for more than 6 days with specific inhibitors of immunoproteasome subunits LMP7 and LMP2 led to a significant downregulation of the expression of pluripotent genes OCT4 and NANOG, as well as to an upregulation of differentiation-associated genes such as BRACHYURY, CDH3, FGF5, NESTIN, PAX6, and GATA4 [17]. However, we found no effect of ONX-0914, a specific inhibitor of Lmp7, on pluripotent markers Oct4 and Nanog in mouse naïve ESCs and EpiLCs (Figure S4a). Also, we did not detect any changes in the expression of formative/primed pluripotency marker Oct6 in mouse EpiLCs treated with ONX-0914, suggesting a normal onset of the formative pluripotency state.

On the other hand, the publicly available transcriptome dataset [30] demonstrated the expression of immunoproteasome subunit Lmp7 into fully primed mouse EpiSCs, but not in EpiLCs (Figure S2). Our results clearly show that immunoproteasome activation during the transition of naïve into primed state already occurs at the formative stage. Although EpiLCs are morphologically and transcriptionally similar to EpiSCs, these two cell types are not identical. The key property of formative pluripotency cells is their ability to give rise to primordial germ cells (PGCs)—a capacity which is lost in the primed pluripotency state [9]. Given this, one would speculate that the immunoproteasomes may play a role in germline specification.

In differentiated cells, such as MEFs, PA28 augments the ability of immunoproteasome to selectively eliminate oxidized proteins [37]. Based on these results, it is tempting to speculate that core immunoproteasome, possibly in complex with the PA28 regulator, are important for oxidative stress adaptation. This possibility is intriguing in the context of the differentiation of pluripotent cells: compared to MEFs, mouse ESCs retain the ability for rapid self-renewal even under hyperoxia conditions, which indicates an enhanced ability of these cells to withstand oxidative stress [2]. At the same time, another study showed that levels of carbonylated proteins sharply decreased between days 2 and 5 of ESC differentiation induced by LIF withdrawal [23]. The level of advanced glycation end products also decreased during differentiation induction. Apparently, the activity of immunoproteasomes and PA28 regulator is increased during the process. Mouse ESCs are known to contain augmented levels of carbonylated proteins and glycation end products; however, according to some studies, such damage is effectively repaired upon differentiation in vitro [23]. Logically, it can be assumed that the immunoproteasomes are involved in these processes. Indeed, experiments with a proteasome-specific inhibitor indicate that proteasomal activity is required to maintain an acceptable level of carbonylated proteins during early ESC differentiation [18]. These results also imply that increased immunoproteasome activity may address the need for the elimination of oxidatively damaged proteins, which occurs at the first signs of cell fate specification. We suppose that it could be critical for cells to approach the formative stage of mouse ESC differentiation with minimal defects at the level of their proteome. However, our preliminary data showed that the inhibition of Lmp7 by treatment with ONX-0914 did not change the level of carbonylated proteins in mouse naïve ESCs and EpiLCs (Figure S4b).

We have found that EpiLCs have an increased proliferation rate compared to naïve ESCs (Figure S3b). On the other hand, a cell proliferation increase requires augmented protein biosynthesis to maintain cell size and functionality. Furthermore, it is known that cells with increased protein synthesis are more sensitive to proteotoxic stress. To cope with this stress, cells activate the unfolded protein response (UPR) in which the UPS plays a central role [38]. Accordingly, increasing the protein degradation is one of the strategies for preventing proteotoxic stress within cells. Apart from the role of immunoproteasomes in peptide generation for MHC-I antigen presentation, the main function of any proteasome complex within cells is to maintain protein homeostasis. Hence, we speculate that the observed upregulation of immunoproteasomes might have evolved to cope with an increased proteolyical demand in EpiLCs.

Recent studies have identified a link between energy metabolism and the epigenetic control of cell state transitions and suggested the stabilization of transient cellular states through metabolic modulation. It has been revealed that the generation of alpha–ketoglutarate (αKG) by the mitochondrial TCA cycle enzyme IDH2 can modulate epigenome, which is involved in the maintenance of naïve pluripotency [39]. The transition to a mostly glycolytic metabolism during EpiLC differentiation, in turn, limits the IDH2-mediated conversion of mitochondrial citrate to αKG, leading to a gradual reduction in intracellular αKG levels. The interrelation between mitochondrial dysfunction or metabolic changes and proteostasis is also interesting in the context of the functional role of immunoproteasomes [40]. It was demonstrated that, in human cells, mitochondrial dysfunction leads to the upregulation of immunoproteasome subunit PSMB9. It can be assumed that this mechanism represents a protective reaction aimed at maintaining cellular proteostasis upon mitochondrial stress. Taken together, one can hypothesize that the activation of immunoproteasomes during the transition from one pluripotent state to another may be associated with metabolic rearrangements and the need for the rapid regulation of proteostasis [41].

Importantly, mice deficient in one, two, or all three immunoproteasome subunits developed normally, except that they showed abnormal antigen processing, CD8+ T-cell responses, and T-cell development [42,43,44,45,46]. This fact implies that either the function of the immunoproteasomes is not required for self-renewal or the differentiation of pluripotent epiblast during embryonic development, or that the loss of immunoproteasome function is compensated by other components of the ubiquitin–proteasome system. Yet another possibility is that immunoproteasome functions become required during embryonic development under the conditions of stress. Supporting this idea, it has been recently shown that the interferon regulatory factor 1, Irf1, is transiently upregulated during the transition from naïve to formative pluripotency [47]. The authors showed that Irf1-deficient mouse EpiLCs were more susceptible to viral infection. Intriguingly, Ifr1 has been shown to mediate the IFNγ-induced upregulation of Lmp7 and the concerted expression of immunoproteasome subunits [48]. Thus, it is tempting to speculate that the susceptibility of Irf1-deficient EpiLCs to viral infection is a consequence of immunoproteasome function loss. Moreover, transcriptomic data showed a decreased expression of immunoproteasome genes in EpiLCs lacking Irf1 [47]. As for the early development, it is possible is that immunoproteasomes have a protective function during the transition of naïve epiblast cells into the primed state, which is coincident with the implantation of the embryo into the uterus. Our preliminary data show that the inhibition of Lmp7 by treatment with ONX-0914 increases the efficiency of the viral infection of ESCs (Figure S4c).

## 5. Conclusions

Investigating molecular mechanisms controlling the transition from naïve to differentiated cells via the formative and primed states of pluripotency is important for better understanding early development, as well as for the efficient and safe introduction of pluripotent cells in regenerative medicine. Investigating the biological functions of immunoproteasomes during the differentiation of pluripotent cells, as well as in the process of pluripotency acquisition via reprogramming, seems to be a highly relevant pursuit. The data presented in this manuscript suggest that immunoproteasome expression is established as early as during the formative pluripotency phase of epiblast development.

## Data Availability

The datasets used and/or analyzed during the current study are available from the corresponding author upon reasonable request.

## Supplementary Materials

The following supporting information can be downloaded at: https://www.mdpi.com/article/10.3390/cells13161362/s1, Table S1: List of antibodies used in Western blot and immunohistochemistry analyses. Table S2: Sequences of primers used for real-time quantitative (qPCR) analysis. Figure S1: The analysis based on the publicly available GSE140890 dataset. Figure S2: GEO2 analysis based on the publicly available GSE30056 dataset. Figure S3: Western blot analysis of several proteasome subunits in naïve ESCs and EpiLC, and the relative growth rate of naïve ESCs and EpiLCs. Figure S4: the influence of ONX-0914 on expression of Oct4, Nanog, and Oct6, on levels of carbonylated proteins in naïve ESCs and EpiLCs, and on viral infections in ESCs.

## References

[1] Thomson J.A., Itskovitz-Eldor J., Shapiro S.S., Waknitz M.A., Swiergiel J.J., Marshall V.S., Jones J.M. (1998). Embryonic stem cell lines derived from human blastocysts. Science.

[2] Saretzki G., Armstrong L., Leake A., Lako M., von Zglinicki T. (2004). Stress defense in murine embryonic stem cells is superior to that of various differentiated murine cells. Stem Cells.

[3] Vilchez D., Simic M.S., Dillin A. (2014). Proteostasis and aging of stem cells. Trends Cell Biol..

[4] Budenholzer L., Cheng C.L., Li Y., Hochstrasser M. (2017). Proteasome Structure and Assembly. J. Mol. Biol..

[5] Maupin-Furlow J.A., Humbard M.A., Kirkland P.A., Li W., Reuter C.J., Wright A.J., Zhou G. (2006). Proteasomes from structure to function: Perspectives from Archaea. Curr. Top. Dev. Biol..

[6] Boes B., Hengel H., Ruppert T., Multhaup G., Koszinowski U.H., Kloetzel P.M. (1994). Interferon gamma stimulation modulates the proteolytic activity and cleavage site preference of 20S mouse proteasomes. J. Exp. Med..

[7] Ebstein F., Kloetzel P.M., Kruger E., Seifert U. (2012). Emerging roles of immunoproteasomes beyond MHC class I antigen processing. Cell. Mol. Life Sci..

[8] Kloetzel P.M., Soza A., Stohwasser R. (1999). The role of the proteasome system and the proteasome activator PA28 complex in the cellular immune response. Biol. Chem..

[9] Smith A. (2017). Formative pluripotency: The executive phase in a developmental continuum. Development.

[10] Gordeev M., Bakhmet E., Tomilin A. (2021). Pluripotency Dynamics during Embryogenesis and in Cell Culture. Russ. J. Dev. Biol..

[11] Buckley S.M., Aranda-Orgilles B., Strikoudis A., Apostolou E., Loizou E., Moran-Crusio K., Farnsworth C.L., Koller A.A., Dasgupta R., Silva J.C. (2012). Regulation of pluripotency and cellular reprogramming by the ubiquitin-proteasome system. Cell Stem Cell.

[12] Choi J., Baek K.H. (2018). Cellular functions of stem cell factors mediated by the ubiquitin-proteasome system. Cell. Mol. Life Sci..

[13] Podenkova U., Zubarev I., Tomilin A., Tsimokha A. (2023). Ubiquitin-Proteasome System in the Regulation of Cell Pluripotency and Differentiation. Cell Tissue Biol..

[14] Vilchez D., Boyer L., Morantte I., Lutz M., Merkwirth C., Joyce D., Spencer B., Page L., Masliah E., Berggren W.T. (2012). Increased proteasome activity in human embryonic stem cells is regulated by PSMD11. Nature.

[15] Noormohammadi A., Calculli G., Gutierrez-Garcia R., Khodakarami A., Koyuncu S., Vilchez D. (2018). Mechanisms of protein homeostasis (proteostasis) maintain stem cell identity in mammalian pluripotent stem cells. Cell. Mol. Life Sci..

[16] Okita Y., Nakayama K.I. (2012). UPS delivers pluripotency. Cell Stem Cell.

[17] Atkinson S.P., Collin J., Irina N., Anyfantis G., Kyung B.K., Lako M., Armstrong L. (2012). A putative role for the immunoproteasome in the maintenance of pluripotency in human embryonic stem cells. Stem Cells.

[18] Hernebring M., Fredriksson A., Liljevald M., Cvijovic M., Norrman K., Wiseman J., Semb H., Nystrom T. (2013). Removal of damaged proteins during ES cell fate specification requires the proteasome activator PA28. Sci. Rep..

[19] Morgani S., Nichols J., Hadjantonakis A.-K. (2017). The many faces of Pluripotency: In vitro adaptations of a continuum of in vivo states. BMC Dev. Biol..

[20] Hanna J., Cheng A.W., Saha K., Kim J., Lengner C.J., Soldner F., Cassady J.P., Muffat J., Carey B.W., Jaenisch R. (2010). Human embryonic stem cells with biological and epigenetic characteristics similar to those of mouse ESCs. Proc. Natl. Acad. Sci. USA.

[21] Takashima Y., Guo G., Loos R., Nichols J., Ficz G., Krueger F., Oxley D., Santos F., Clarke J., Mansfield W. (2014). Resetting transcription factor control circuitry toward ground-state pluripotency in human. Cell.

[22] Theunissen T.W., Powell B.E., Wang H., Mitalipova M., Faddah D.A., Reddy J., Fan Z.P., Maetzel D., Ganz K., Shi L. (2014). Systematic identification of culture conditions for induction and maintenance of naive human pluripotency. Cell Stem Cell.

[23] Hernebring M., Brolen G., Aguilaniu H., Semb H., Nystrom T. (2006). Elimination of damaged proteins during differentiation of embryonic stem cells. Proc. Natl. Acad. Sci. USA.

[24] Murphy C.L., Polak J.M.J.T.e. (2002). Differentiating embryonic stem cells: GAPDH, but neither HPRT nor β-tubulin is suitable as an internal standard for measuring RNA levels. Tissue Eng..

[25] Veazey K.J., Golding M.C.J.P.o. (2011). Selection of stable reference genes for quantitative rt-PCR comparisons of mouse embryonic and extra-embryonic stem cells. PLoS ONE.

[26] Wang X., Chen C.F., Baker P.R., Chen P.L., Kaiser P., Huang L. (2007). Mass spectrometric characterization of the affinity-purified human 26S proteasome complex. Biochemistry.

[27] Endoh M., Niwa H. (2022). Stepwise pluripotency transitions in mouse stem cells. EMBO Rep..

[28] Lauria A., Meng G., Proserpio V., Rapelli S., Maldotti M., Polignano I.L., Anselmi F., Incarnato D., Krepelova A., Donna D. (2023). DNMT3B supports meso-endoderm differentiation from mouse embryonic stem cells. Nat. Commun..

[29] Kim I.S., Wu J., Rahme G.J., Battaglia S., Dixit A., Gaskell E., Chen H., Pinello L., Bernstein B.E. (2020). Parallel single-cell RNA-seq and genetic recording reveals lineage decisions in developing embryoid bodies. Cell Rep..

[30] Hayashi K., Ohta H., Kurimoto K., Aramaki S., Saitou M. (2011). Reconstitution of the mouse germ cell specification pathway in culture by pluripotent stem cells. Cell.

[31] Ferrington D.A., Gregerson D.S. (2012). Immunoproteasomes: Structure, function, and antigen presentation. Prog. Mol. Biol. Transl. Sci..

[32] Heink S., Ludwig D., Kloetzel P.M., Kruger E. (2005). IFN-gamma-induced immune adaptation of the proteasome system is an accelerated and transient response. Proc. Natl. Acad. Sci. USA.

[33] Rock K.L., Gramm C., Rothstein L., Clark K., Stein R., Dick L., Hwang D., Goldberg A.L. (1994). Inhibitors of the proteasome block the degradation of most cell proteins and the generation of peptides presented on MHC class I molecules. Cell.

[34] Mishto M., Liepe J., Textoris-Taube K., Keller C., Henklein P., Weberruß M., Dahlmann B., Enenkel C., Voigt A., Kuckelkorn U. (2014). Proteasome isoforms exhibit only quantitative differences in cleavage and epitope generation. Eur. J. Immunol..

[35] Groettrup M., Ruppert T., Kuehn L., Seeger M., Standera S., Koszinowski U., Kloetzel P.M. (1995). The interferon-gamma-inducible 11 S regulator (PA28) and the LMP2/LMP7 subunits govern the peptide production by the 20 S proteasome in vitro. J. Biol. Chem..

[36] van den Eshof B.L., Medfai L., Nolfi E., Wawrzyniuk M., Sijts A.J. (2021). The function of immunoproteasomes—An immunologists’ perspective. Cells.

[37] Pickering A.M., Davies K.J. (2012). Differential roles of proteasome and immunoproteasome regulators Pa28alphabeta, Pa28gamma and Pa200 in the degradation of oxidized proteins. Arch. Biochem. Biophys..

[38] Qu J., Zou T., Lin Z. (2021). The roles of the ubiquitin–proteasome system in the endoplasmic reticulum stress pathway. Int. J. Mol. Sci..

[39] Tischler J., Gruhn W.H., Reid J., Allgeyer E., Buettner F., Marr C., Theis F., Simons B.D., Wernisch L., Surani M.A. (2019). Metabolic regulation of pluripotency and germ cell fate through α-ketoglutarate. EMBO J..

[40] Kim M., Serwa R.A., Samluk L., Suppanz I., Kodroń A., Stępkowski T.M., Elancheliyan P., Tsegaye B., Oeljeklaus S., Wasilewski M. (2023). Immunoproteasome-specific subunit PSMB9 induction is required to regulate cellular proteostasis upon mitochondrial dysfunction. Nat. Commun..

[41] Sinenko S.A., Tomilin A.N. (2024). Metabolic control of induced pluripotency. Front. Cell Dev. Biol..

[42] Fehling H.J., Swat W., Laplace C., Kuhn R., Rajewsky K., Muller U., von Boehmer H. (1994). MHC class I expression in mice lacking the proteasome subunit LMP-7. Science.

[43] Basler M., Moebius J., Elenich L., Groettrup M., Monaco J.J. (2006). An altered T cell repertoire in MECL-1-deficient mice. J. Immunol..

[44] Van Kaert L., Ashton-Rickardt P.G., Eichelberger M., Gaczynska M., Nagashima K., Rock K.L., Goldberg A.L., Doherty P.C., Tonegawa S. (1994). Altered peptidase and viral-specific T cell response in LMP2 mutant mice. Immunity.

[45] Kincaid E.Z., Che J.W., York I., Escobar H., Reyes-Vargas E., Delgado J.C., Welsh R.M., Karow M.L., Murphy A.J., Valenzuela D.M. (2012). Mice completely lacking immunoproteasomes show major changes in antigen presentation. Nat. Immunol..

[46] Caudill C.M., Jayarapu K., Elenich L., Monaco J.J., Colbert R.A., Griffin T.A. (2006). T cells lacking immunoproteasome subunits MECL-1 and LMP7 hyperproliferate in response to polyclonal mitogens. J. Immunol..

[47] Romeike M., Spach S., Huber M., Feng S., Vainorius G., Elling U., Versteeg G.A., Buecker C. (2022). Transient upregulation of IRF1 during exit from naive pluripotency confers viral protection. EMBO Rep..

[48] Namiki S., Nakamura T., Oshima S., Yamazaki M., Sekine Y., Tsuchiya K., Okamoto R., Kanai T., Watanabe M. (2005). IRF-1 mediates upregulation of LMP7 by IFN-γ and concerted expression of immunosubunits of the proteasome. FEBS Lett..

