# Supplementary material for: Evidence of Immunoproteasome Expression Onset in the Formative State of Pluripotency in Mouse Cells
Source: Cells. 2024 Aug 15;13(16):1362. doi: 10.3390/cells13161362 (PMC11352244; doi:10.3390/cells13161362)
Supplement: Supplementary file 1 [file cells-13-01362-s001.zip › cells-3067854-supplementary.pdf]

Supplementary Materials

Table S1. List of antibodies used in Western blot and immunohistochemistry analyses

**Primary antibody**		
Rpn7	Enzo Life Sciences PW8225	1:1000
α7	Enzo Life Sciences PW8110	1:1000
Rpn1 (PSMD2)	ServiceBio GB113525	1:1000
Lmp7	Kindly provided by Prof. Dr. Ulrike Seifert (University Medicine Greifswald, Germany)	1:5000
PA28α	Cell Signaling #2408	1:1000
PA28β	Cell Signaling #2409	1:1000
β-Actin	Cell Signaling #3700	1:5000
Oct4	Santa Cruz sc-5279	1:500
Nanog	Bethyl A300-397 or Cell Signaling #8822	1:500
α-Tubulin	Sigma T6074	1:5000
**Secondary antibodies**		
HRP-GAR	Jackson Immunoresearch 111-036-003	1:5000
HRP-GAM	Jackson Immunoresearch 115-036-003	1:5000
HRP-GAGP	Jackson Immunoresearch 106-036-003	1:5000
Alexa Fluor® 647-GAR	Jackson Immunoresearch 111-606-003	1:500
Alexa Fluor® 488-GAM	Jackson Immunoresearch 115-546-003	1:500

Table S2. Sequences of primers used for real-time quantitative (qPCR) analysis

Gene	Primer sequence (5'-3')	
Pou5f1 (Oct4)	F<br>R	AGTGGAAAGCAACTCAGAGG<br>AACTGTTCTAGCTCCTTCTGC
Klf4	F<br>R	TACCCCTACACTGAGTCCCG<br>GGAAAGGAGGGTAGTTGGGC
Nanog	F<br>R	GCTCCATAACTTCGGGGAGG<br>GTGCTAAAATGCGCATGGCT
Zfp42 (Rex1)	F<br>R	CCCTCGACAGACTGACCCTAA<br>TCGGGGCTAATCTCACTTTCAT
Esrrb	F<br>R	GTCTGACACTTGGGGACCAG<br>CTACCAGGCGAGAGTGTTCC
Otx2	F<br>R	ACTTGCCAGAATCCAGGGTG<br>CTTCTTCTTGGCAGGCCTCA
Pou3f1 (Oct6)	F<br>R	AGTTCGCCAAGCAGTTCAAG<br>TGGTCTGCGAGAACACGTTA
Fgf5	F<br>R	AAAGTCAATGGCTCCCACGAA<br>GGCACTTGCATGGAGTTTTCC
Psma4 (α3)	F<br>R	TCCCTGTGAGCAGTTGGTTAC<br>GCTTATCCCAGCCAATATACAGC
Psma5 (α5)	F<br>R	GAGTACGACAGGGGTGTGAAT<br>GCTCCATTAGTGGGGAGGTAA
Psma7 (α4)	F<br>R	CCTTGGACGATAACGTCTGTATG<br>ACTCGCAATGTAGCGGGTG
Psmb5 (β5)	F<br>R	CCACAGCAGGTGCTTATATTGC<br>GCTCATAGATTCGACACTGCC
Psmb6 (β1)	F<br>R	GCCTTAGCTGTTCGTCGAG<br>TAGAACCACGCCCCCATTAAA
Psmb7(β2)	F<br>R	GTGTCGGTGTTTCAGCCAC<br>GTGCCAGTTTTCCGAGCTTTC
Psmb8 (β5i/Lmp7)	F<br>R	GTGCAGGTTGTATTATCTTCGGA<br>CGAGTCCCATTGTCATCTACG
Psmb9 (β1i/Lmp2)	F<br>R	AGGAGCACCTACCGCCG<br>AGAGGGGAGAGCTTGTCGAAC
Psmb10 (β2i/Mecl-1)	F<br>R	GAGGAATGCGTCCTTGGAACA<br>CACAACCGAATCGTTAGTGGC
Psmc1 (Rpt2)	F<br>R	GATAGGGGTGCTAATGGATGACA<br>GCTTTATCCCCATCTCCTCGTA
Psmc3 (Rpt5)	F<br>R	AGCTACCAACCATGTCACTCC<br>CTTCAAGGATCGCATCACACA
Psmd1 (Rpn2)	F<br>R	CAGGCTACAAAGGAAACCATTGA<br>CCCAGTGTGAAGGCTCTCT
Psmd3 (Rpn3)	F<br>R	CAGGCTACCCTTTTGAACCTC<br>GGCCCACTCATTGTTGTTGG
Psmd6 (Rpn7)	F<br>R	GGCTTATATTGTGTGGCTATCCG<br>CTGAGGTCCGGCCTTTCTAA
Psmd14 (Rpn11)	F<br>R	TGTAAACACAATGAATCGGTGGT<br>CGGCCAAACACTGGACAAT

Psme1 (Pa28α)	F<br>R	GACATCCCAGTACCCGATCCA<br>GTAGCTGCAACCAGGTAGTGA
Psme2 (Pa28β)	F<br>R	CTAAGTGTGGCTACCTCCCG<br>TCCATGTGATTACCAGAATGCAC
Psmg1 (Pac1)	F<br>R	GTCTGGGAAGAAGTCGGTTGT<br>CCGAGGGATCTGATTTCAGTTG
Psmg2 (Pac2)	F<br>R	TGCCAGGATCATTGTTCTCTCA<br>GATGCACCGACTCTTTTCCAT
Psmg3 (Pac3)	F<br>R	TGACCCAGTTCGGGAAGATG<br>GACGGCTCTGTTTCCTGCTT
Psmg4 (Pac4)	F<br>R	GACGTGTCGCTTCACAACTTC<br>ACTGCGAGGTTGCGTAGATG
Pomp	F<br>R	AGGTTCACCGTCTCCCGTT<br>TCACCCATCAGTTCACTTTGTG
Gapdh	F<br>R	AGGTCGGTGTGAACGGATTTG<br>TGTAGACCATGTAGTTGAGGTCA
B2m (Beta-2-microglobulin)	F<br>R	ACCCGCCTCACATTGAAATCC<br>GGCGTATGTATCAGTCTCAGTG

Continuation of Table S2.

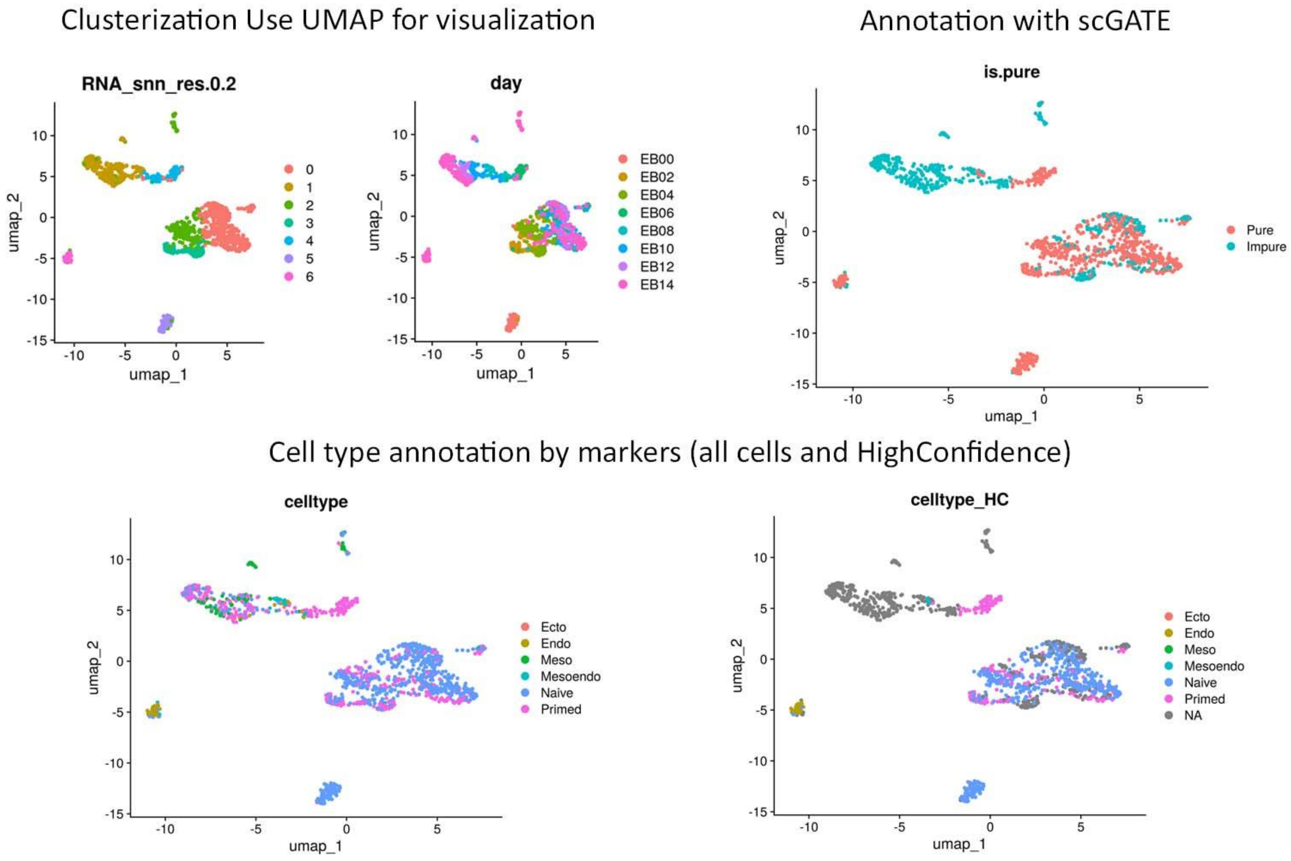

Figure S1. scRNA-seq profiling of differentiating mouse EBs based on publicly available dataset GSE140890 in The Gene Expression Omnibus (Kim et al., 2020; doi: 10.1016/j.celrep.2020.108222).

The Seurat pipeline was employed to normalize and perform cell cycle scoring, followed by scaling by regression of cell cycle scores to minimize their impact on downstream analyses. Principal component analysis (PCA) was conducted using the top 20 principal components, followed by UMAP (Uniform Manifold Approximation and Projection) for dimensionality reduction and visualization. Clustering was performed by Louvain clustering algorithm using a resolution of 0.2. Then clusters were visualized on UMAP plots. Cell type annotation was performed using scGate with curated list of markers for various cell states. The expression of proteasome-related genes (Psmb8, Psmb9, Psmb10, and Psma5) was analyzed across different clusters and time points.

Proteasome gene expression

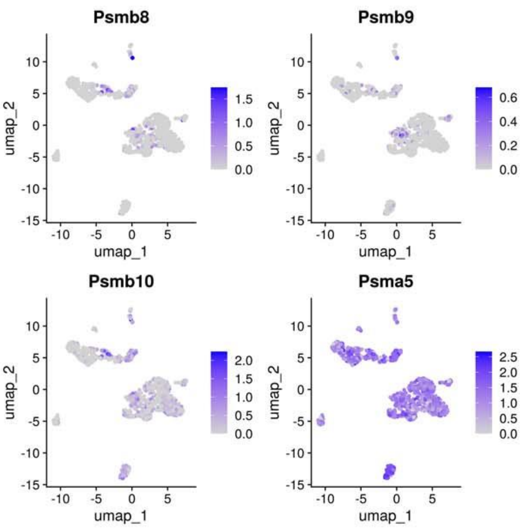

ViolinPlot on celltypes

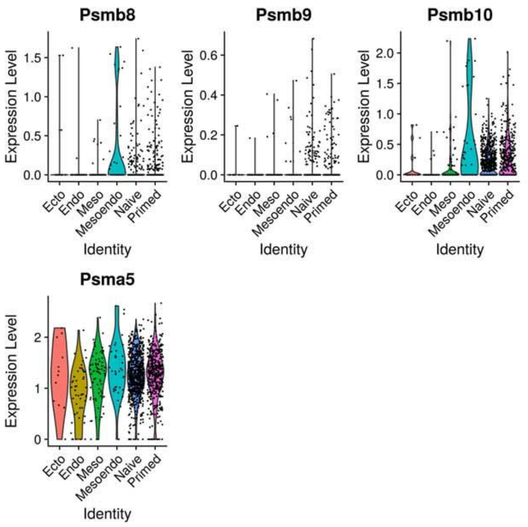

ViolinPlot on celltypes HighConfidence
(NA - not annotated)

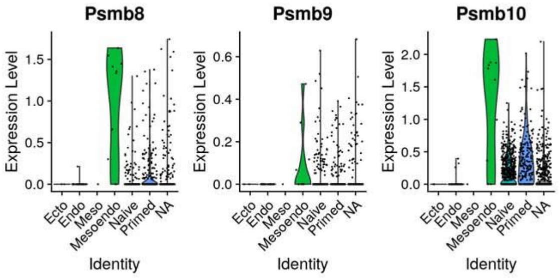

Continuation of Figure S1.

Psmb8

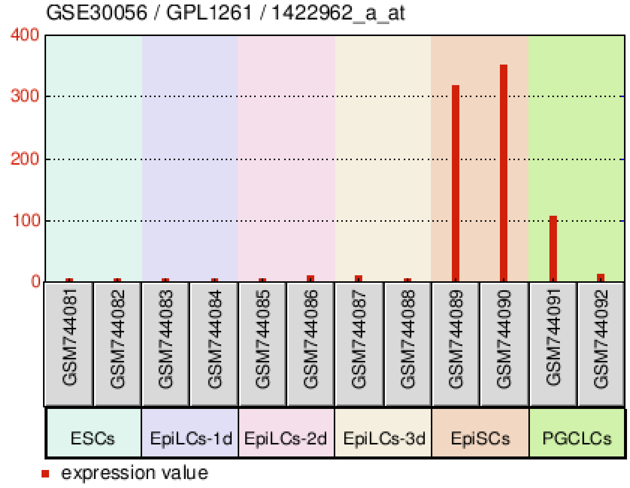

Psmb9

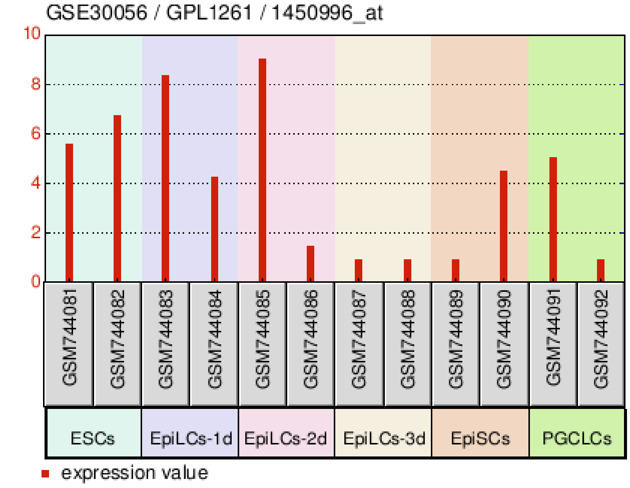

Psmb10

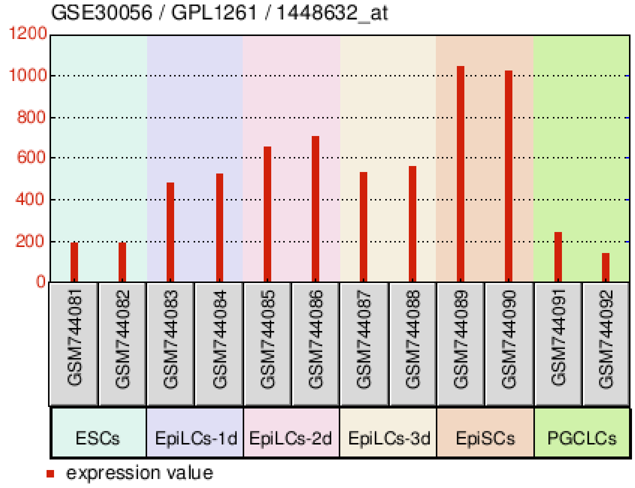

GSM744081	embryonic stem cells (ESCs) (2iLIF, feeder free), biological rep1
GSM744082	embryonic stem cells (ESCs) (2iLIF, feeder free), biological rep2
GSM744083	day 1 epiblast like cells (EpiLCs), biological rep1
GSM744084	day 1 epiblast like cells (EpiLCs), biological rep2
GSM744085	day 2 epiblast like cells (EpiLCs), biological rep1
GSM744086	day 2 epiblast like cells (EpiLCs), biological rep2
GSM744087	day 3 epiblast like cells (EpiLCs), biological rep1
GSM744088	day 3 epiblast like cells (EpiLCs), biological rep2
GSM744089	epiblast stem cells (EpiSCs), biological rep1
GSM744090	epiblast stem cells (EpiSCs), biological rep2
GSM744091	primordial germ cell (PGC) like cells (PGCLCs), biological rep1
GSM744092	primordial germ cell (PGC) like cells (PGCLCs), biological rep2

Figure S2. Expression of immunoproteasome subunits genes in ESCs, EpiLCs, EpiSCs, and PGCLCs. Figure generated by GEO2-analyzer, based on publicly available dataset GSE30056 in The Gene Expression Omnibus (Hayashi et al., 2011; doi:10.1016/j.cell.2011.06.052).

Psmb8

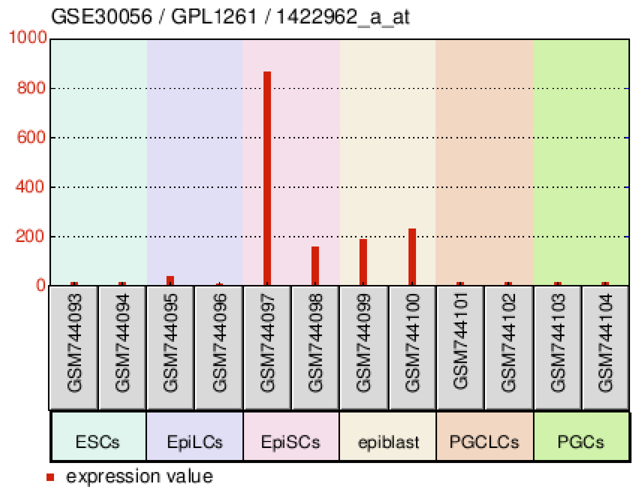

Psmb9

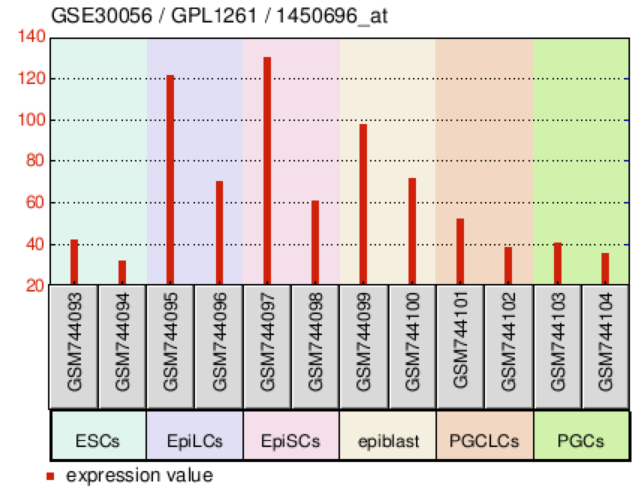

Psmb10

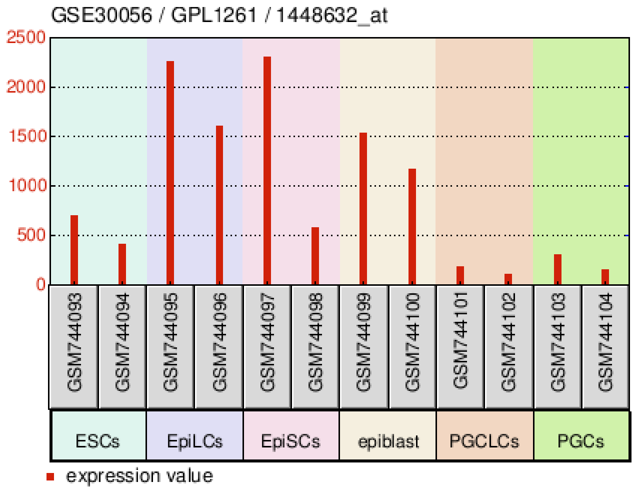

GSM744091	primordial germ cell (PGC) like cells (PGCLCs), biological rep1
GSM744092	primordial germ cell (PGC) like cells (PGCLCs), biological rep2
GSM744093	embryonic stem cells (ESCs), PCR amplified, biological rep1
GSM744094	embryonic stem cells (ESCs), PCR amplified, biological rep2
GSM744095	day 2 epiblast like cells (EpiLCs), PCR amplified, biological rep1
GSM744096	day 2 epiblast like cells (EpiLCs), PCR amplified, biological rep2
GSM744097	epiblast stem cells (EpiSCs), PCR amplified, biological rep1
GSM744098	epiblast stem cells (EpiSCs), PCR amplified, biological rep2
GSM744099	epiblast, PCR amplified, biological rep1
GSM744100	epiblast, PCR amplified, biological rep2
GSM744101	PGCLCs, PCR amplified, biological rep1
GSM744102	PGCLCs, PCR amplified, biological rep2
GSM744103	PGCs (E9.5), PCR amplified, biological rep1
GSM744104	PGCs (E9.5), PCR amplified, biological rep2

Continuation of Figure S2.

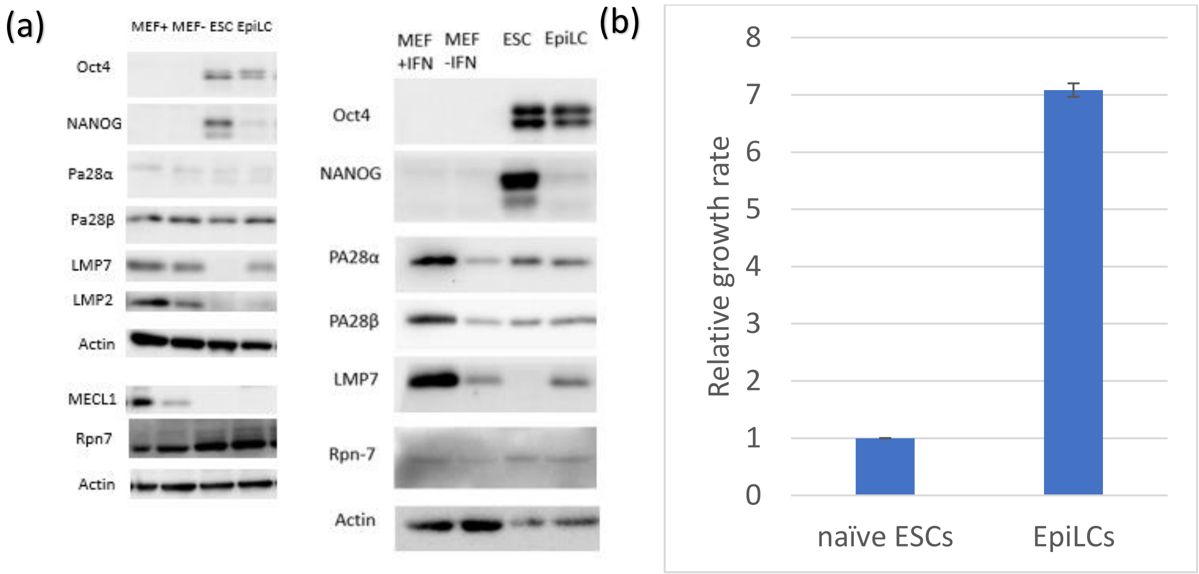

Figure S3. The transition of ESCs from the naive to the formative pluripotency state (featured by EpiLCs) induces the expression of immunoproteasome subunit Lmp7 is also associated with an increased proliferation rate of EpiLCs compared to naive ESCs.

a) Western blot analysis of immunoproteasome subunit Lmp7, PA28 regulator subunits α and β, 19S regulator subunit Rpn7, Oct4 and Nanog (pluripotency markers) in naive ESCs and EpiLCs lysates, MEF treated with IFNγ served as a positive control.

b) Growth rate of naïve ESCs and EpiLCs. Data are from biological triplicates with mean ± s.d.

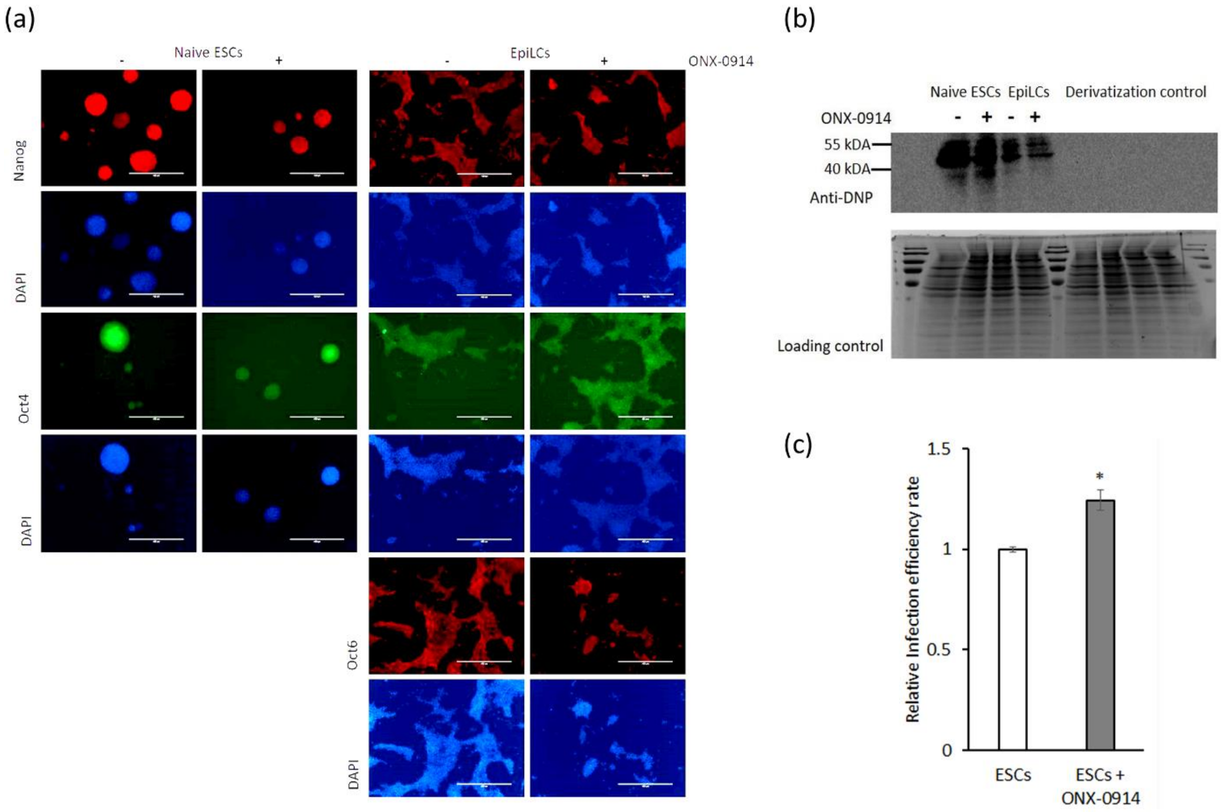

Figure S4. Inhibition of Lmp7 by a specific inhibitor, ONX-0914 (100 nM), have no effect on Oct4, Nanog, and Oct6 as well as levels of carbonylated proteins in naïve ESCs and EpiLCs. However, it does lead to an increase in viral infection in ESCs.

a) Representative immunostaining of naive ESCs (left panel) and EpiLCs (right panel) with or without ONX-0914 treatment (+ or -, respectively), using antibodies against Oct4 (green), Nanog (red), or Oct6 (red). Nuclei were counterstained with DAPI (blue). Scale bar – 400 µm;

b) Monitoring protein carbonylation in response to ONX-0914 treatment in naive ESCs and EpiLCs by OxyBlot Protein Oxidation Detection Kit. DNPH labeled (or unlabeled, as `Derivatization control` here) proteins presented on anti-DNP blot (top). The residual proteins in the gel were visualized by Coomassie staining (bottom).

c) Quantification of GFP+ cells by FACS analysis after retro virus (pQCXIP-GFP) infection. Values are shown as means ± s.e.m. of at least three independent experiments. Significant values are indicated as *$p < 0.05$ (one-way ANOVA followed by Tukey's post hoc test).
